# Microsatellite (SSR) amplification by PCR usually led to polymorphic bands: Evidence which shows replication slippage occurs in extend or nascent DNA strands

**Published:** 2016-09

**Authors:** Abasalt Hosseinzadeh-Colagar, Mohammad Javad Haghighatnia, Zahra Amiri, Maryam Mohadjerani, Majid Tafrihi

**Affiliations:** Department of Molecular and Cell Biology, Faculty of Basic Sciences, University of Mazandaran, Babolsar, Mazandaran, Iran

**Keywords:** Microsatellites, *Taq* polymerase slippage, Replication slippage

## Abstract

Microsatellites or simple sequence repeats (SSRs) are very effective molecular markers in population genetics, genome mapping, taxonomic study and other large-scale studies. Variation in number of tandem repeats within microsatellite refers to simple sequence length polymorphism (SSLP); but there are a few studies that are showed SSRs replication slippage may be occurred during *in vitro *amplification which are produced ‘stutter products’ differing in length from the main products. The purpose of this study is introducing a reliable method to realize SSRs replication slippage. At first, three unique primers designed to amplify SSRs loci in the great gerbil (*Rhombomys opimus*) by PCR. Crush and soak method used to isolate interesting DNA bands from polyacrylamide gel. PCR products analyzed using by sequencing methods. Our study has been shown that *Taq *DNA polymerase slipped during microsatellite *in vitro *amplification which led to insertion or deletion of repeats in sense or antisense DNA strands. It is produced amplified fragments with various lengths in gel electrophoresis showed as ‘stutter bands’. Thus, in population studies by SSRs markers recommend that replication slippage effects and stutter bands have been considered.

## INTRODUCTION

Microsatellites or simple sequence repeats (SSRs) are short repetitive elements of 1- 6 bases that found in prokaryotic and all eukaryotic genomes [1]. These repeat motifs, which present in both coding and noncoding regions [2, 3] are smaller than 100bp [4]. Microsatellites are highly polymorphic, reproducible, abundant, inherited co-dominantly and distribute throughout genome [1, 5]. The excessive rate of mutation, high number of alleles and frequency in the genomic DNA, have made SSRs very effective molecular markers in population genetics, genome mapping, taxonomic study, linkage analysis, genetic fingerprinting and diversity [1, 2, 6-9]. Generation of new alleles and microsatellite instability can be related to several diseases [10]. SSRs can be amplified by the standard polymerase chain reaction (PCR), using specific primer sequences from the fl anking regions [1, 4, 5, 7]. Also, the microsatellites are the best choice for forensics and noninvasive sampling studies because of high sensitivity of PCR [1]. Because of that, these frequently repetitive motifs make microsatellites extremely prone to mutation [11]. Thus microsatellite polymorphisms arise generally from variability in length rather than in the primary sequence [2]. Previous studies have shown the replication slippage in amplification of long [12] and short [13] tandem repeats [14]. It also has been observed that a replication slippage occurs during *in vitro *amplification of microsatellite sequences and appears as a minor product that differs in size from the main product called ‘stutter bands’ or ‘shadow bands’ [2]. Other studies showed that the PCR amplification of short tandem repeat (STR) typically produces a minor product band shorter than the main allele band; it’s referred to as ‘stutter band’ [15, 16]. In the present study, we evaluated possible *Taq *polymerase slippage in an SSR polymorphism study in the Great gerbil (*Rhombomys opimus*) as animal sample.

## MATERIALS AND METHODS

**Sample collecting & Blood DNA extraction: **Three Great Gerbils’ (*Rhombomys opimus*) blood samples from Iran population, including: Gonbad-e Kavus, Maraveh Tappeh, Sarakhs (Sangar), Sarakhs (Gonbadli), Esfarayen, Shahrood, Damghan, Natanz and Isfahan which are held in the Molecular and Cell biology laboratory of UMZ (Babolsar, Iran), were used randomly. Genomic DNA was extracted in regard to salting- out procedure [17]. Quality and quantity of isolated DNA was analyzed by Green and Sambrook method [18].

**Primers design & Polymerase chain reaction: **For amplification of SSRs loci, at first three SSR loci of great gerbil, which brought in the NCBI gene bank with HM469957, HM469963 and HM469954 accession numbers, were selected. The HM469957, HM469963 and HM469954 loci included (AC)16, (AC)3 (AG) (CATG)2 (CA)11 and (AC)6 repeat motifs, respectively. Then six specific oligomers, as a primer, were designed by OLIGO version 7.0 software. These primers including: *MAGN*27 (with forward: 5'-CAT GTA TTG GGC AGA TAT ACA TG-3' and reverse: 5'-TTC GAC ACA TAG TTC CTG AAA C-3') for HM469957; *MAGN*78 (with forward: 5'-ATG TTC CCA CTT ATC CTT TCA G-3' and reverse: 5'-CGA TAT CAA GAG ATC AAA AGG-3') for HM469963, and *MAGN*19 (with forward: 5'-ATA AAC AAC AAC TAG CTC TTA G- ' and reverse: 5'-TAA ATC TAT AGG AAC CTT CTA G-3') for HM469954, have been designed. All primers ere ordered by Bioneer Co. (Korea), as a lyophilized. PCR reaction performed in 50 µl final volumes with mixture of 25 ng of genomic DNA, 5µl 10X PCR buffer, 0.5 mM mix dNTP, 1.5mM MgCl2, 0.5mM of each forward and reverse primers and 0.2 U of *Taq *DNA polymerase. The amplification figure consisted of an initial denaturation for 5 minutes at 94°C followed by 35 cycles of 1 minute at 94°C, 30 seconds at the annealing temperature (57°C for *MAGN27*; 63°C for *MAGN78 *and 51°C for *MAGN19*), 1 minutes for elongation at 72°C, and a final extension step of 5 minutes at 72°C.

**Gel electrophoresis: **The genomic DNA was analyzed by 1% agarose gel electrophoresis and stained by RedSafe™ (Intron co. Korea). The PCR products were electrophoresed on 8% polyacrylamide gel (30.8 % acrylamide bisacrylamide) in TBE buffer (25 mM Tris, 25 mM Boric acid, 50 mM EDTA, pH 8.0) at 180 W for 2-3hours, depending on the fragment sizes. Then, the polyacrylamide gel stained by DNA silver staining (AgNO3) methods [18]. All of the electrophoresis materials provided from Merck Company.

**Polyacrylamide gel DNA isolation & Sequencing: **The target DNA bands on the polyacrylamide gel were extracted by modified crush and soak method [18]. Summarily, the target bands were cut out from gels, and then sliced into small pieces. Subsequently, they transferred the small pieces to a sterile tube and added 2 volumes of elution buffer (0.5 M Ammonium acetate and 10mM Magnesium acetate and 1 mM EDTA pH 8.0). Then incubate at 37°C overnight, on a rotating wheel. After that, samples were centrifuged at 12000 rpm for 10 minutes at 20°C. The supernatant was recovered. Finally, the extracted DNA was precipitated with 2.5 volume absolute ethanol. These isolated PCR products were sequenced by Bioneer Co. (Korea) and analyzed by Chromas software ver 2.4.

## RESULTS

**Figure1:**
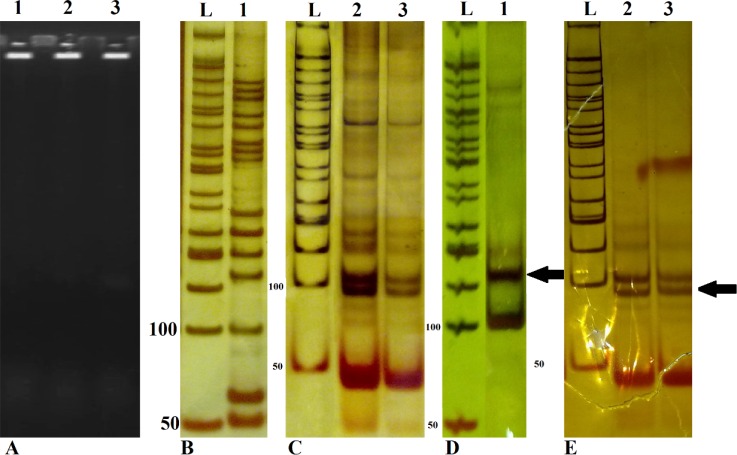
The genomic DNA and PCR products in agarose and polyacrylamide (PA) gel electrophoresis:

**Figure 2:**
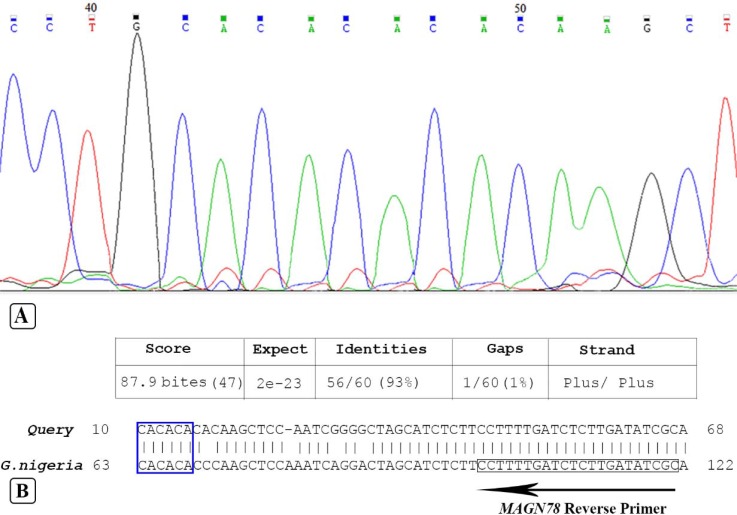
Electropherogram and alignment results: A) Chromatogram results of MAGN78 fragments isolated by MAGN78 reverse primer; B) Our query sequence align using BLAST (bl2seq); Reverse primer region indicate by arrow-box; The repeat sequences numbers in the *G. nigeriae *and *R. opimus *indicate by blue-box; New nucleotide insertion indicated by small arrow

The extracted DNA electrophoresed in 1% agarose gel, results showed that genomic DNA was acceptable for PCR (Fig. 1a). SSRs loci fragments amplified by PCR technique and electrophoresed in PAGE. We showed many bands as nonspecific (Fig. 2b) that are very confusing with calculated size of our interesting bands. Because we expected that designed flanking primers, based on NCBI gene bank with HM469957, HM469963 and HM469954 accession numbers, amplify about 97, 116 and 106bp, respectively. In another study, to confirm these fragments, target bands isolate by crush and soak method. Then isolated fragments re-amplified by PCR. These re-amplified products detected by PAGE. In spite of our expectation, result of stained gels showed the same nonspecific bands (Fig. 1c).

In this figure, which electrophoresis was performed on 8% PAGE, different base pair (9bp) between line 2 (MAGN19) and line 3 (MAGN27) didn't observe but after sequencing these difference was detectable. Analysis of PCR directed sequences performed by compared to tow PCR sequences: sequence results of the PCR products which crush and soak from PCR fragments, directly and renew-PCR. Our result showed tow insertions in sequencing of renew-PCR. In contrast, we didn’t observe any insertion from the PCR fragments, which amplified from genomic DNA. We conclude that these insertions occurred because of taq slippage. And amplification of SSRs by PCR contains a (CA) repeat sequence (Fig. 2a). The alignment of these sequences with NCBI data base sequence confirmed tow deletion repeat sequence courted for example *MANG78*, too (Fig. 2b).

## DISCUSSION

The study of genetic changes and analysis of microsatellite in the genome is more difficult than other common sequences. PCR was used for amplification of microsatellite. This technique can increase the rate of mutations in the genome by errors during amplification that is known as PCR noise [19]. One of the errors that may occur in the amplification process of these sequences is known as polymerase slippage [20]. High rate of this error observe in single-nucleotide repeat regions [21]. Although the molecular mechanism of the described errors during amplification of repetitive DNA sequence motifs, are not well established, they are the great interest during analysis and diagnosis of various diseases. For example, mononucleotide microsatellite sequences are hotspots for mammalian polymerase error during *in vivo *DNA replication. This process of mutation is called as slipped strand mispairing (SSM).

**Figure 3:**
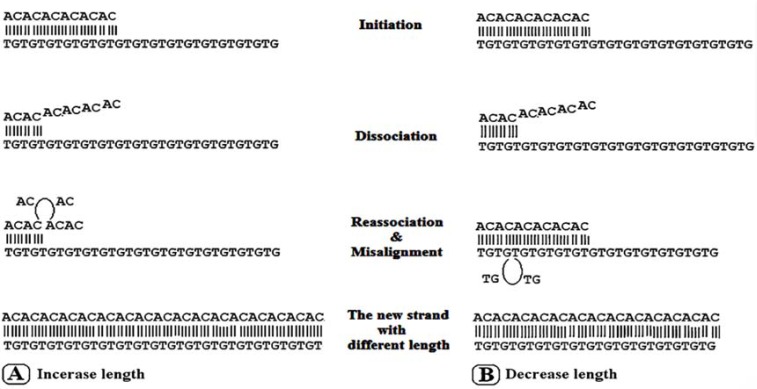
The mechanism of replication slippage from (AC)n repeats: A) In increasing length, repeats bulge in the extending DNA strands. B) In decreasing length repeats bulge in nascent DNA strands

In this study, at first we observed PCR products of SSRs loci in gel electrophoresis. Then amplified SSR target bands isolated by crush and soak method, which are different from nonspecific bands, and re-amplified (Fig. 1). Insertion or deletion of repeats may be occurred. Fazekas *et al*. (2010), reported that mutations at SSR sites during *in vitro *enzymatic replication of SSRs are usually the result of insertion or deletion of repeats in the extending or nascent DNA strands [11]. Altering microsatellite length derived from a mutation mechanism that is specific to tandemly repeated sequences that are called replication slippage [22]. During synthesis the repetitive region two strands can disassociate. Sense strands might incorrectly realign with repeat units. Ellegren predicted that if incorrect alignment creates a loop upstream on the sense strand, the result would be a raise in repeat length; and if the incorrect alignment occurs upstream on the antisense strand, a loop that is formed in the antisense strand causes a decrease in repeat length [2, 22] (Fig. 3). Analysis of isolated PCR products by sequencing confirmed *Taq *DNA polymerase slippage during SSRs *in vitro *amplification.

The results obtained using different DNA polymerases appear to support the slipped strand mutation as a potential explanation for how these stutter products are generated [15, 16]. Sequencing of PCR products has been shown the main cause of ‘stutter bands’ in the PCR with normal conditions is changing in the number of repeat units due to replication slippage of *Taq *DNA polymerase [21]. Slippage might take place either in the active site of the enzyme or before the substrate binds to the enzyme [23]. Replication slippage has been occurring efficiently during the first replication cycle of PCR [14]. Based on our findings, it existence some of the nonspecific bands which may be results of *Taq *slippages in general or specific PCR. A researcher can be removed these bands by altering PCR conditions, but removing of all nonspecific bands in microsatellite amplification and some of the sequence repeats such as VNTR by *Taq *DNA polymerase is inevitable.
